# Long-term high-dose l-arginine supplementation in patients with vasculogenic erectile dysfunction: a multicentre, double-blind, randomized, placebo-controlled clinical trial

**DOI:** 10.1007/s40618-021-01704-3

**Published:** 2022-01-01

**Authors:** D. Menafra, C. de Angelis, F. Garifalos, M. Mazzella, G. Galdiero, M. Piscopo, M. Castoro, N. Verde, C. Pivonello, C. Simeoli, R. S. Auriemma, A. Colao, R. Pivonello

**Affiliations:** 1 Dipartimento di Medicina Clinica e Chirurgia, Sezione di Endocrinologia, Unità di Andrologia e Medicina della Riproduzione e della Sessualità Maschile e Femminile (FERTISEXCARES), Università Federico II di Napoli, Via Sergio Pansini 5, 80131 Naples, Italy; 2 Unesco Chair for Health Education and Sustainable Development, Federico II University, Naples, Italy

**Keywords:** l-arginine, Vasculogenic erectile dysfunction, Nitric oxide, Sexual function, Penile duplex ultrasonography, PDE5i

## Abstract

**Purpose:**

The current randomized, double-blind, placebo-controlled clinical trial addressed the effects on penile erectile function of relatively high daily oral doses (6 g/day) of l-ARG for 3 months (*N* = 51) compared to placebo (*N* = 47), in patients with vasculogenic ED, with comparison between mild–moderate and severe vasculogenic ED.

**Methods:**

The outcome measures included IIEF-6 score and cavernous arteries peak systolic flow velocity (PSV) at dynamic penile duplex ultrasonography (PDU).

**Results:**

l-ARG supplementation for 3 months significantly increased IIEF-6 score in the overall cohort (*p* < 0.0001) and in subgroups of patients with mild–moderate (*p* < 0.0001) and severe (*p* = 0.007) vasculogenic ED; PSV was significantly increased in the overall cohort (*p* < 0.0001) and in patients with mild–moderate (*p* < 0.0001), but not severe vasculogenic ED. At study completion, 74% of patients improved ED degree category, although only 24% of patients, mainly belonging to the baseline category of mild ED, reached IIEF-6 scores compatible with absence of ED; moreover, 20% of patients, exclusively belonging to the baseline category of mild–moderate vasculogenic ED, reached PSV values compatible with absence of ED.

**Conclusion:**

The results of the current study demonstrated that supplementation with relatively high doses of l-ARG as a single compound for 3 months significantly improved penile erectile function, assessed by both IIEF-6 score and PSV at dynamic PDU in patients with mild–moderate, and improved IIEF-6 score, but not PSV, in patients with severe vasculogenic ED, therefore suggesting that l-ARG might be an alternative treatment in mild–moderate vasculogenic ED patients experiencing adverse effects or with contraindications for chronic treatment with PDE5i compounds.

## Introduction

Penis erection, or tumescence, is the physiological process of spontaneous or sexually induced enlargement and hardening of the penis, as a result of a complex interaction of psychological, neural, vascular, and endocrine factors [1]. The first trigger of penis tumescence derives from the peripheral nervous system, which induces, through the inhibition of sympathetic and the stimulation of parasympathetic activity, the relaxation of the smooth muscle belonging either to the wall of the arterial system, which flows into the typical lacunar spaces, or to the trabecular structure, which delimitates the lacunar spaces, of the corpora cavernosa; the smooth muscle relaxation leads to cavernous arteries vasodilation and cavernous lacunar spaces extension, with consequent increase of blood inflow into the corpora cavernosa of the penis [1]. The engorgement of cavernous lacunar spaces induces a compression of the cavernous venous system, with consequent decrease of blood outflow from the corpora cavernosa, ultimately entrapping the blood into the penis and maintaining penis tumescence [1]. The process is reversed by the inhibition of parasympathetic and the stimulation of sympathetic activity, which induces the contraction of the cavernous smooth muscle, leading to a decrease of blood inflow, and the consequent gradual decompression of the cavernous venous system, leading to an increase of blood outflow, ultimately inducing the passage from tumescence to detumescence of the penis, till the achievement of penis flaccidity, a condition characterized by a tonic contraction of the cavernous smooth muscle allowing a small amount of arterial blood flow for nutritional purpose [1].

The process of cavernous smooth muscle relaxation, which is crucial for penis erection, is not only due to sympathetic adrenergic inhibition and parasympathetic cholinergic stimulation, but predominantly induced by the concomitant activation of the nitrergic system and the consequent production of nitric oxide (NO), an important mediator of smooth muscle relaxation [1]. In particular, the activation of the parasympathetic cholinergic neurons determines the release of acetylcholine, which stimulates NO production by the cavernous arteries endothelial NO synthase (eNOS), whereas the activation of the parasympathetic non-adrenergic non-cholinergic (NANC) neurons determines NO production by the NANC neuronal NO synthase (nNOS) [1]. The mechanism of action of NO is based on the activation in the cavernous smooth muscle of the enzyme guanylate cyclase, responsible for the generation of the cyclic GMP (cGMP),  activating a molecular signalling pathway determining a decrease of intracellular calcium concentration and consequently the cavernous smooth muscle relaxation, which is at the basis of the penis tumescence [1]. The process is reversed by the enzyme phosphodiesterase type 5 (PDE5), which induces the cGMP hydrolysis, determining an increase of intracellular calcium concentration and consequently the cavernous smooth muscle contraction, which is at the basis of penis detumescence, testifying PDE5 to be a crucial enzyme in the reversion of penis erectile process and in maintenance of penis flaccid state [1]. It is noteworthy that penis tumescence is modulated by psychological conditions, as well as by the endocrine system, particularly by the androgenic status, which essentially exerts a permissive role for the penile erectile function; indeed, testosterone displays a positive action on desire and sexual function and specifically contributes, through the enhancing of the activity of penile NOS enzymes, to the cavernous smooth muscle relaxation and ultimately to penis erection [2, 3].

Erectile dysfunction (ED) results from pathological derangement of the crosstalk among the nervous, vascular and smooth muscle systems, involved in the regulation of penis tumescence and detumescence processes [4, 5]. ED is a common pathological condition affecting male sexual activity, and is defined as the inability to attain or maintain a penis erection sufficient for a successful intercourse [5]. The prevalence of ED increases with age and is therefore much higher in elderly than in young men, but still relatively frequent during middle age; the prevalence rates are estimated to range from 1 to 15% and from 6 to 40% in men aged 30–50 and 50–80 years, respectively, whereas a 50 to 100% prevalence is estimated in men older than 70 years of age [6]. The etiology of ED comprises psychogenic and organic diseases, the latter being determined by vascular, neurological, or endocrine disorders, as well as pharmacological factors, which could also occur simultaneously; vasculogenic ED is the most common form of organic ED, and is determined by the reduction of cavernous blood inflow, consequence of arterial insufficiency, generally induced by vascular disease and, particularly, endothelial dysfunction [4, 7]. The diagnosis of ED is made on the basis of clinical and andrological history and examination, scoring derived by the International Index of Erectile Function questionnaire (IIEF), as well as performance of penile duplex ultrasonography (PDU) in flaccid status (basal PDU) and/or pharmacologically induced erectile status (dynamic PDU) [4].

The oral PDE5 inhibitors (PDE5i) currently represent the first-line treatment for ED [8–10]. PDE5i inhibit cGMP degradation and increase penile cavernous smooth muscle relaxation, therefore prolonging the effects of cGMP to potentiate the erection [10–12]. Although the great efficacy of PDE5i has been widely demonstrated for the treatment of ED, regardless of the etiology, some factors might limit their employment; in particular, the generally favourable safety profile is limited by contraindications and/or the occurrence of adverse effects, whereas the relevant efficacy is limited by partial or complete resistance in a subgroup of patients, and lastly, the relatively high cost might have a negative impact on the chronic use of this category of drugs; these factors contribute to treatment discontinuation [7–13]. On the other hand, several nutraceuticals, including yohimbine, ginseng, niacin, l-carnitine and l-arginine (l-ARG), supplemented as single agents and/or in different combinations, have been reported to offer benefits in the treatment of ED, without adverse effects and with the additional advantage to have a more affordable cost [14, 15]. Taking into account the concept that nutraceuticals are considered safer and are generally less costly than PDE5i, these agents might represent a valid therapeutic alternative in the treatment of ED, particularly for the treatment of non-severe or at least mild ED, or ED unresponsive to PDE5i, or in case of intolerance to PDE5i.

l-ARG, a conditionally essential amino acid introduced by dietary proteins and produced in the body from the amino acid l-citrulline (l-CIT), has been recognized as a potential candidate in the treatment of ED, since it represents the physiological substrate for NO biosynthesis [7, 16, 17]. Indeed, NO is synthesized from l-ARG and oxygen by the nNOS and eNOS enzymes, in the neurons of cavernous NANC fibres and arteries endothelial cells, respectively, with release of l-CIT, which can be reconverted into l-ARG, therefore fueling a further NO-producing cycle [18]. Noteworthy, the potential role of l-ARG supplementation is corroborated by the evidence that a significant proportion of patients with ED, particularly of vasculogenic etiology, are characterized by a decrease of NO production in the penile vascular endothelium, such as in the case of ED caused by diabetes and atherosclerosis [16, 17, 19], and by low l-ARG or l-CIT levels, compared to men without ED, therefore suggesting that low levels of these amino acids might increase the risk of ED by inducing the reduction of NO availability [20].

Taking into consideration that NO plays a crucial role as mediator of penile erectile function and l-ARG represents the physiological precursor for the penile NO biosynthesis, research has been focused on l-ARG supplementation by yielding promising results in the treatment of ED [7]. A meta-analysis of 10 randomized clinical trials (RCTs) with l-ARG supplementation, as single agent or in combination with additional compounds, demonstrated that supplementation with l-ARG at a daily dose ranging from 2.8 to 8 g, with supplementation schedules from 2 weeks to 6 months, significantly improved mild–moderate ED or overall ED of unspecified severity, compared to placebo-treated or untreated patients, with ED heterogeneously assessed by different tools comprising subjective and/or validated questionnaires, including IIEF, and results being consistent across different dosage and duration of l-ARG supplementation; conversely, a very low dose (1.5 g) of l-ARG administered daily for a very short time (17 days) was ineffective [7].

The current study aimed at unequivocally addressing the effects of a relatively high-dose l-ARG supplementation, administered as single compound in a long-term treatment schedule, on penile erectile function in a large cohort of male patients with vasculogenic ED, with comparison of response between mild–moderate to severe vasculogenic ED.

## Patients and methods

### Study design

The current study is a randomized, double-blind, placebo-controlled clinical trial on the effects of a 3-month l-ARG supplementation on penile erectile function in male patients with vasculogenic ED. The main outcome of the study was the penile erectile function assessed by IIEF 6-item (IIEF-6) score and cavernous arteries peak systolic flow velocity (PSV) obtained at dynamic PDU. A secondary aim of the study was to detect differential responses to l-ARG supplementation according to the degree of baseline vasculogenic ED assessed at dynamic PDU. At study entry, patients were allocated to l-ARG or placebo group using standard randomization tables; patients and clinicians were blinded regarding the treatment modality. Intervention schedule included a 3-month treatment with l-ARG (6 g/day), administered orally thrice a day after standard meals, using vials containing 2 g l-ARG/20 ml (Bioarginina^®^, Farmaceutici Damor S.p.A., Napoli, Italy), or placebo. Daily l-ARG supplementation regimen was established according to current l-ARG administration schedule used in clinical practice and, particularly, to l-ARG administration schedule used in interventional studies performed in patients with ED. l-ARG and placebo were provided in vial packaging of the same color, shape, and size. At study entry, participants received 160 vials in 8 packs containing 20 vials (135 bottles for treatment and 25 as a reservoir). At 45 days after study entry, excess of unused vials was withdrawn, and patients received additional 160 vials in 8 packs containing 20 vials (135 bottles for treatment and 25 as a reservoir) for treatment completion, occurring at 90 days after study entry. The evaluation of patients was performed at two time-points, namely at study entry or baseline (T0), and at study completion or end of treatment (T1). At T0 and T1 the assessment of penile erectile function was performed by IIEF-6 questionnaire and dynamic PDU, and associated with the registration of medical, pharmacological and sexual anamnesis, a complete physical examination, comprising the measurement of clinical parameters [height, weight, body mass index (BMI), heart rate (HR), and systolic (SBP) and diastolic (DBP) blood pressure], as well as a blood collection, performed in the morning and after an overnight fast, for the evaluation of biochemical parameters [fasting glucose (FG), triglycerides (TG), total and HDL cholesterol, and indexes of renal and liver function], for either the exclusion of confounding factors or safety. An endocrine evaluation with the measurement of the most important hormones involved in the regulation of sexual function (total testosterone and prolactin) was performed at baseline to exclude the main endocrine disorders affecting penile erectile function; total testosterone levels were also re-tested at study completion. The occurrence of adverse events was recorded during the entire study and up to 15 days after study completion; adverse events were scored as mild (well tolerated and not interfering with daily activities), moderate (poorly tolerated but not interfering with daily activities) or severe (determining death, impairment of vital functions, disability, hospitalization). The study was performed in line with the principles of the Declaration of Helsinki, after the approval by a local Ethics Committee.

### Patients

The current study enrolled patients with vasculogenic ED following the inclusion and exclusion criteria, in the respect of confidentiality and anonymity, ensured by assigning participants a code number for the purpose of analysis. Inclusion criteria were represented by the presence at the study entry of the following conditions: (1) age 20–75 years; (2) stable sexual relationship; (3) mild to moderate ED assessed by the IIEF-6 score, not attributable to psychological, neurological or endocrinological factors, concomitant diseases or pharmacological treatment, which were excluded by clinical interview, and attributable exclusively to a vasculogenic etiology, which was confirmed at dynamic PDU on the basis of cavernous arteries PSV < 35 cm/s; (4) normal serum total testosterone and prolactin levels. Exclusion criteria were represented by the presence at the study entry of the following conditions: (1) use of vasodilatory medications, particularly medications increasing NO production; (2) use of drugs potentially impairing penile erectile function; (3) recent (less than 6 months) cardiovascular (CV) or cerebrovascular events and/or unstable hemodynamic conditions; (4) severe systemic or organ diseases; (5) history of radical pelvic surgery; (6) psychiatric disorders and/or treatment for psychiatric disorders; (7) alcoholism or suspicion of alcohol abuse; (8) use or abuse of addictive substances.

One-hundred adult Caucasian patients with age range of 20–73 years with vasculogenic ED were recruited during 5 consecutive years, after the achievement of an informed consent for the participation to the study. Among the 100 patients enrolled in the study, 2 patients voluntarily discontinued at an early stage of the study, before randomization to the treatment arm and starting treatment, and were not included in the study analysis of the overall cohort, which therefore definitely considered 98 patients: 51 patients were assigned to l-ARG group and 47 patients were assigned to placebo group. Considering the overall cohort of 98 patients included in the final study analysis, 3 patients discontinued before reaching T1, by determining a 3.06% drop-out rate at study completion and a final overall cohort of 95 patients evaluated at all time-points. In particular, 1 patient in l-ARG group discontinued before reaching T1, by determining a 1.96% drop-out rate at study completion and a final group of 50 patients; conversely, in placebo group, 2 patients discontinued before reaching T1, by determining a 4.26% drop-out rate at study completion and a final group of 45 patients. Twenty-three (23.47%) patients enrolled in the study presented with concomitant diseases, including hypercholesterolemia (1), hypertension (15), glucose intolerance (1), diabetes (3), hypothyroidism (1), prostatic hypertrophy (1), diverticulitis (1).

### Assessment of penile erectile function

Penile erectile function was assessed by the administration of IIEF-6 questionnaire, with the registration of IIEF-6 score, and the performance of a dynamic PDU, with the registration of the cavernous arteries PSV.

#### IIEF-6 questionnaire

IIEF-6 questionnaire was administered through face-to-face interview performed by trained personnel; patients were asked to provide response to six questions and a total IIEF-6 score between 1 and 30 points was obtained from the IIEF-6 scoring system. According to this scoring system, a score within the range 26–30 points indicates absence of ED, whereas a score < 26 points indicates ED; mild ED is defined by a score within 22–25 points, mild–moderate ED within 17–21 points, moderate ED within 11–16 points, severe ED ≤ 10 points.

#### Dynamic PDU

Dynamic PDU was performed by specifically trained and experienced clinicians; in order to avoid inter-operator variability, the same clinician performed both the  T0 and T1 evaluations for a given patient. Dynamic PDU was performed according to standard procedures, using an ultra-sonographer device, equipped with linear, high‐resolution, and high‐frequency (7.5 to 14 MHz) probes, with color Doppler for detecting slow flow and a scanning surface of at least 5 cm. Before PDU, 10 µg of PGE1 (Alprostadil, Caverject, Pfizer S.r.l., Latina, Italy) was injected laterally into corpora cavernosa at the distal two‑third of the penis using a syringe with a 30‑gauge needle under aseptic condition. PDU was then performed every 10 min after pharmacological injection, for 20–30 min; in particular, longitudinal and transverse penile scans were evaluated on both grey scale and color Doppler studies, by placing the probe on the ventral surface of the penis. The waveforms were obtained alternately using an angle of inclination equal or below 60° to obtain an optimal visualization of cavernous arteries. PSV was sampled bilaterally in right and left cavernous arteries, considering the lower value for the analysis of the study; a PSV < 35 cm/s indicated arterial disease, and, therefore the presence of vasculogenic ED; mild–moderate vasculogenic ED was defined as a PSV comprised between 25 cm/s and 35 cm/s whereas severe vasculogenic ED was defined as a PSV < 25 cm/s.

### Statistical analysis

Statistical analysis was performed with GraphPad Prism and IBM SPSS Statistics softwares. Continuous variables with normal distribution were reported as mean ± standard deviation (SD), whereas non-normally distributed variables were reported as median with interquartile range (25°–75° centiles). Within-group changes at different time-points in l-ARG or placebo group were analyzed by paired Student’s *t *test for normally distributed variables or Wilcoxon test for variables following non-normal distribution. Differences between l-ARG and placebo groups were analyzed with unpaired Student’s *t *tests for normally distributed variables or Mann–Whitney test for variables following non-normal distribution. Within-group and between-groups comparisons of outcome measures and relative changes in outcome measures were performed in the overall cohort, and separately in subgroups of patients with baseline mild–moderate or severe vasculogenic ED assessed at dynamic PDU, to detect differential responses to the intervention, according to the severity of baseline vasculogenic ED. Categorical variables were reported as absolute numbers and percentages. Difference between l-ARG and placebo groups relative to the prevalence of mild, mild–moderate and moderate ED assessed by IIEF-6 questionnaire, as well as to the prevalence of mild–moderate and severe vasculogenic ED assessed at dynamic PDU was analyzed with Chi-squared test; baseline difference between l-ARG and placebo groups relative to the prevalence of concomitant diseases was analyzed with Fisher’s exact test. A two-tailed *p* value < 0.05 was considered as significant.

## Results

### Baseline cohort characteristics

Ninety-eight patients (20–73 years) were recruited for the study and randomized in l-ARG or placebo group; 51 (20–73 years) patients were allocated to l-ARG group whereas 47 (27–71 years) patients were allocated to placebo group.

At baseline, in patients in l-ARG or placebo group, no significant difference was found regarding clinical and biochemical parameters. No significant difference was found regarding serum total testosterone and prolactin levels, which were within the normal range in the totality of the patients. No significant difference was found in the prevalence of concomitant diseases. The clinical, biochemical and hormonal characteristics, and the prevalence of concomitant diseases of the patients’ population at baseline are shown in Table 1.

**Table 1:** Clinical, biochemical and hormonal characteristics, and prevalence of concomitant diseases of patients’ population at baseline

	l-arginine (*N* = 51)	Placebo (*N* = 47)	*p* value
Age (years)	50 ± 14 (20–73)	53 ± 10 (27–71)	NS
Height (cm)	173 ± 6	173 ± 5	NS
Body weight (kg)	77 ± 9	79 ± 9	NS
BMI (kg/m^2^)	25.8 ± 3	26.2 ± 2.7	NS
Heart rate (bpm)	73 ± 9	73 ± 8	NS
Systolic blood pressure (mmHg)	136 ± 17	134 ± 12	NS
Diastolic blood pressure (mmHg)	80 ± 7	78 ± 9	NS
Fasting glucose (mg/dl)	102 ± 0.2	106 ± 18	NS
Triglycerides (mg/dl)	161 ± 97	159 ± 62	NS
Total cholesterol (mg/dl)	196 ± 37	213 ± 37	NS
HDL cholesterol (mg/dl)	57.9 ± 18.8	58.6 ± 15.4	NS
Urea (mg/dl)	36 ± 9	39 ± 11	NS
Creatinine (mg/dl)	0.9 ± 0.2	0.9 ± 0.2	NS
AST (U/l)	25 ± 9	25 ± 9	NS
ALT (U/l)	29 ± 12	30 ± 15	NS
Total testosterone (nmol/l)	17.7 ± 5.6	19.9 ± 4.4	NS
Prolactin (µg/l)	11 ± 3.3	10.9 ± 3.2	NS
Hypercholesterolemia	–	1	NS
Hypertension	11	4	NS
Glucose intolerance	–	1	NS
Diabetes	3	–	NS
Hypothyroidism (in treatment)	1	–	NS
Prostatic hypertrophy	–	1	NS
Diverticulitis	1	–	NS

Values expressed as mean ± SD, (range), or number of cases

At baseline, in l-ARG or placebo group, no significant difference was found regarding IIEF-6 score [20 (16–22) vs 20 (17–22); *p* = 0.799] and PSV [25.9 (24.6–28) cm/s vs 27.1 (24.2–29.6) cm/s; *p* = 0.055].

**Fig. 1:**
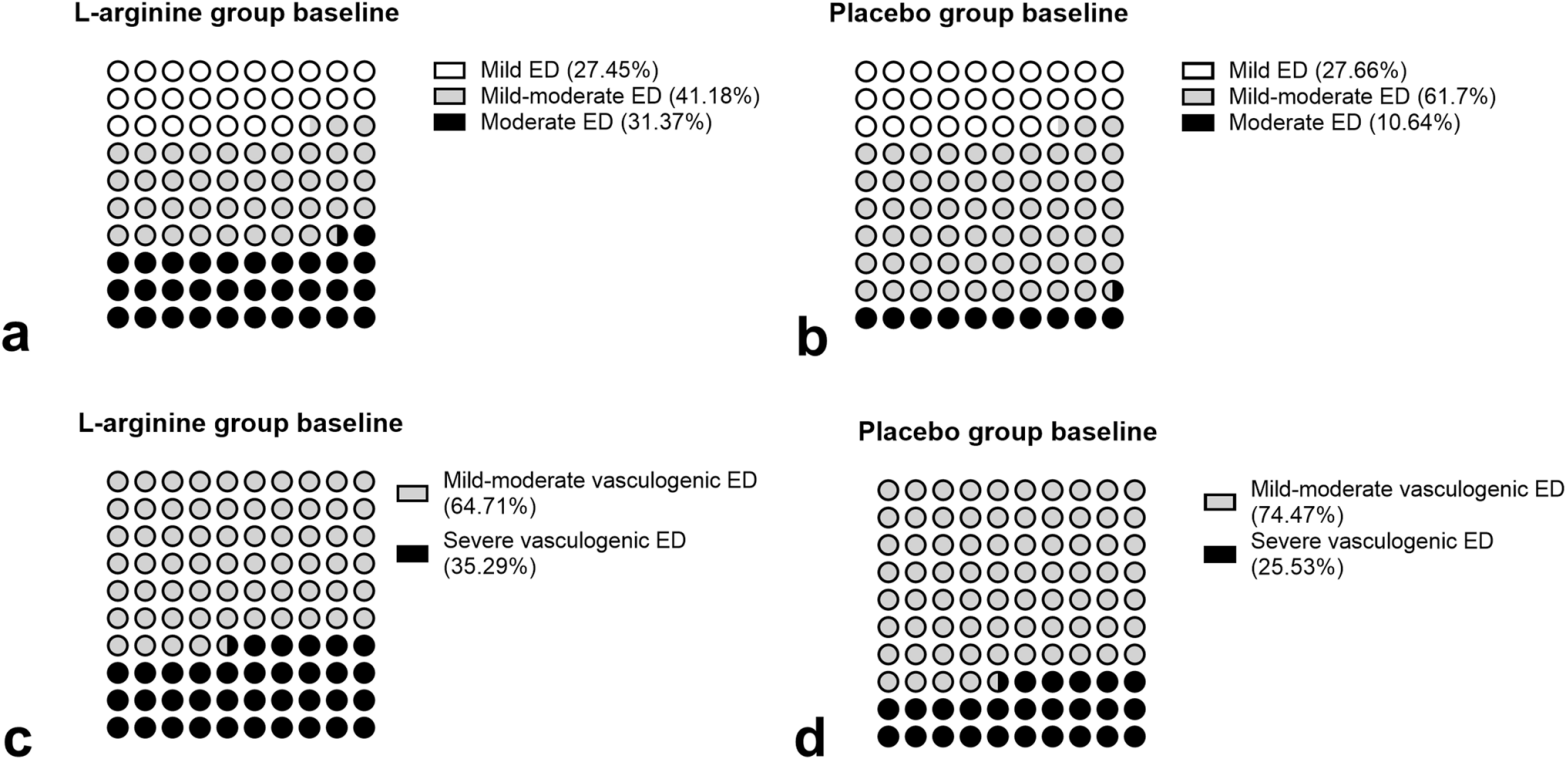
Prevalence of mild (white dots), mild–moderate (gray dots) and moderate (black dots) ED assessed by IIEF-6 questionnaire, in l-arginine (**a**) and placebo (**b**) groups at baseline; prevalence of mild–moderate (gray dots) and severe (black dots) vasculogenic ED assessed at dynamic PDU, in l-arginine (**c**) and placebo (**d**) groups at baseline

In l-ARG group, 14 (27.45%) patients were affected by mild, 21 (41.18%) by mild–moderate and 16 (31.37%) by moderate ED, assessed by IIEF-6 questionnaire; moreover, 33 (64.71%) patients were affected by mild–moderate and 18 (35.29%) by severe vasculogenic ED, assessed at dynamic PDU (Fig. 1). In particular, among the 18 patients with severe vasculogenic ED, 10 had mild–moderate and 8 moderate ED, according to IIEF-6.

In placebo group, 13 (27.66%) patients were affected by mild, 29 (61.7%) by mild–moderate and 5 (10.64%) by moderate ED, assessed by IIEF-6 questionnaire; moreover, 35 (74.47%) patients were affected by mild–moderate and 12 (25.53%) by severe vasculogenic ED, assessed at dynamic PDU (Fig. 1). In particular, among the 12 patients with severe vasculogenic ED, 8 had mild–moderate and 4 moderate ED according to IIEF-6.

In l-ARG or placebo group, no significant difference was found regarding the prevalence of mild ED assessed by IIEF-6 questionnaire, whereas a significantly lower prevalence of mild–moderate (*p* = 0.047) and higher prevalence of moderate (*p* = 0.015) ED was found in l-ARG, compared to placebo group. No significant difference was found regarding the prevalence of mild–moderate and severe vasculogenic ED, assessed at dynamic PDU, between the two groups.

The evaluation of penile erectile function and the prevalence of the different degree category of ED and vasculogenic ED of the patients’ population at baseline are shown in Table 2.

**Table 2:** Evaluation of penile erectile function, and prevalence of mild, mild–moderate and moderate ED assessed by IIEF-6 questionnaire^a^, and prevalence of mild–moderate and severe vasculogenic ED assessed at dynamic PDU^b^, at baseline (T0) and after 90 days (T1), in l-arginine and placebo groups, in the overall cohort

	l-arginine T0 (*N* = 51)	l-arginine T1 (*N* = 50)	*p *value	Placebo T0 (*N* = 47)	Placebo T1 (*N* = 45)	*p *value
IIEF-6 score	20 (16–22)	24 (19–25.3)	< 0.0001	20 (17–22)	20 (17–22)	NS
PSV (cm/s)	25.9 (24.6–28)	30.5 (23.4–34.3)	< 0.0001	27.1 (24.2–29.6)	27.1 (24.5–29.6)	NS
Mild ED^a^	14/51 (27.45%)	18/50 (36%)	NS	13/47 (27.66%)	11/45 (24.44%)	NS
Mild–moderate ED^a^	21/51 (41.18%)	16/50 (32%)	NS	29/47 (61.7%)^§^	23/45 (51.11%)	NS
Moderate ED^a^	16/51 (31.37%)	4/50 (8%)	0.005	5/47 (10.64%)°	10/45 (22.22%)	NS
No ED^a^	0/51 (0%)	12/50 (24%)	0.0001	0/47 (0%)	1/45 (2.23%)	NS
Mild–moderate vasculogenic ED^b^	33/51 (64.71%)	22/50 (44%)	0.047	35/47 (74.47%)	27/45 (60%)	NS
Severe vasculogenic ED^b^	18/51 (35.29%)	18/50 (36%)	NS	12/47 (25.53%)	14/45 (31.11%)	NS
No ED^b^	0/51 (0%)	10/50 (20%)	< 0.001	0/47 (0%)	4/45 (8.89%)	NS

Values expressed as median with interquartile range (25°–75° centiles), or number of cases (%)

*PSV* cavernous arteries peak systolic flow velocity

^§^*p* = 0.047 compared to T0 l-arginine, °*p* = 0.015 compared to T0 l-arginine

In l-ARG or placebo subgroups of patients with mild–moderate vasculogenic ED, no significant difference was found regarding IIEF-6 score [21 (17.5–23.5) vs 21 (19–23); *p* = 0.949], whereas PSV was significantly lower [27.2 (25.9–29.2) cm/s vs 28.2 (27–31.2) cm/s; *p* = 0.020] in l-ARG subgroup; in l-ARG or placebo subgroups of patients with severe vasculogenic ED, no significant difference was found regarding IIEF-6 score [18 (15.8–20.3) vs 17 (15.3–17.8); *p* = 0.248] nor PSV [23.2 (21.8–24.7) cm/s vs 21.5 (19–23.9) cm/s; *p* = 0.131]. The evaluation of penile erectile function of the mild–moderate and severe vasculogenic ED patients’ subpopulations at baseline are shown in Table 3**.**

**Table 3:** Evaluation of penile erectile function at baseline (T0) and after 90 days (T1), in l-arginine and placebo subgroups of patients with baseline mild–moderate or severe vasculogenic ED, assessed at dynamic PDU

	l-arginine T0	l-arginine T1	*p *value	Placebo T0	Placebo T1		*p *value
Mild–moderate vasculogenic ED subgroup IIEF-6 score	21 (17.5–23.5)	25 (21.3–29)	< 0.0001	21 (19–23)	21 (19.8–22)		NS
Mild–moderate vasculogenic ED subgroup PSV (cm/s)	27.2 (25.9–29.2)	33.6 (30.8–35.6)	< 0.0001	28.2 (27–31.2)^§^	28.1 (26.7–31.7)		NS
Severe vasculogenic ED subgroup IIEF-6 score	18 (15.8–20.3)	21 (18–24.3)	0.007	17 (15.3–17.8)	16 (15–17)		NS
Severe vasculogenic ED subgroup PSV (cm/s)	23.2 (21.8–24.7)	22.1 (20.8–23.9)	NS	21.5 (19–23.9)	21 (20–23.7)		NS

Values expressed as median with interquartile range (25°–75° centiles)

*PSV* cavernous arteries peak systolic flow velocity

^§^*p* = 0.020 compared to T0 l-arginine

### Effect of l-ARG supplementation on penile erectile function in the overall cohort

The treatment mean duration was 89.9 vs 91.1 days, in l-ARG and placebo groups, respectively, reflecting the mean number of unused vials returned to the investigators in the two groups; no significant difference in treatment mean duration was detected between the two groups. A non-significant trend towards an increase in total testosterone levels was detected at T1 in l-ARG (17.9 ± 5.6 nmol/l vs 17.7 ± 5.6 nmol/l; *p* = 0.055) group, but not in placebo (19.8 ± 4.3 nmol/l vs 19.9 ± 4.4 nmol/l; *p* > 0.1) group, compared to T0.

#### IIEF-6 questionnaire

**Fig. 2:**
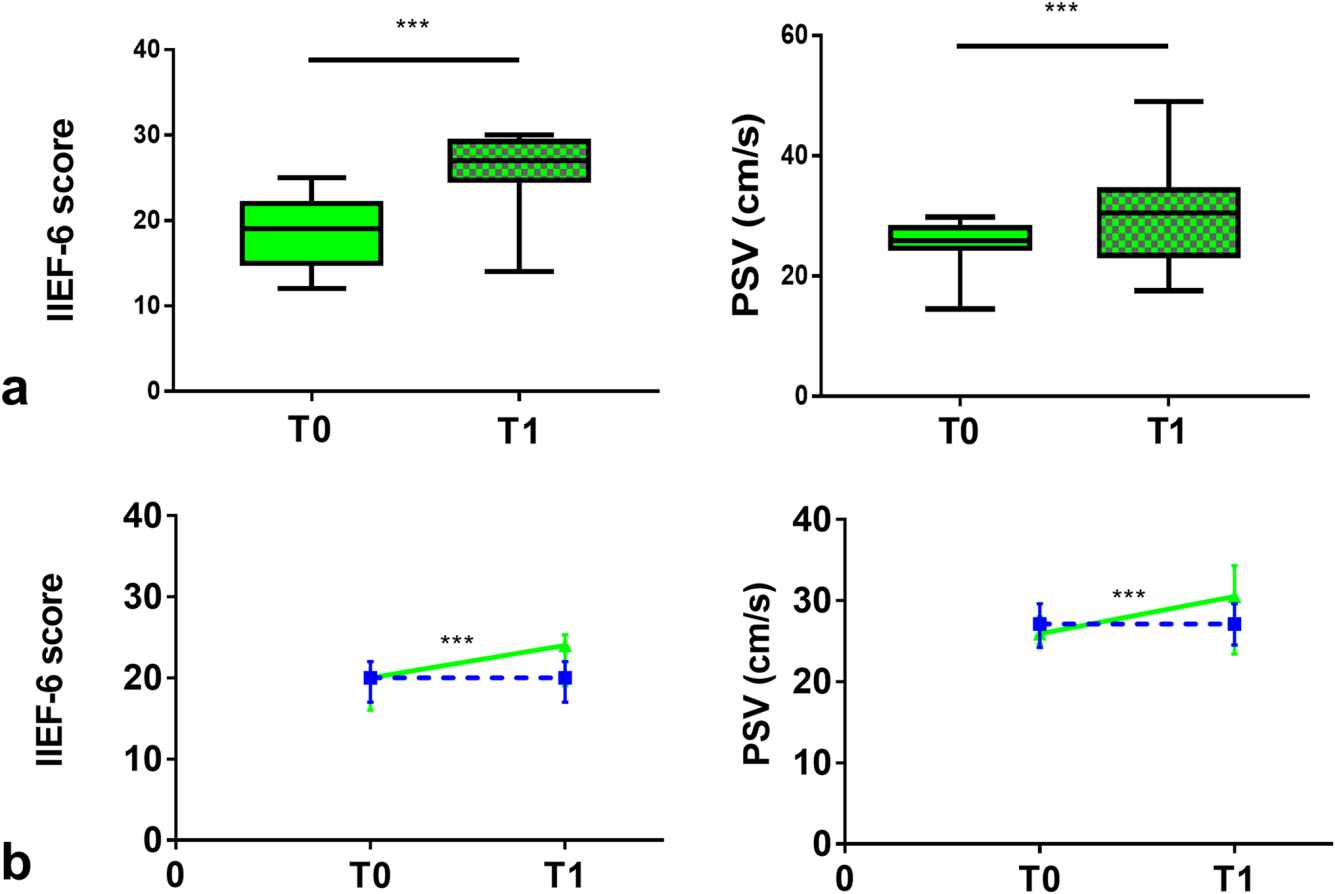
**a** IIEF-6 score and cavernous arteries peak systolic flow velocity (PSV), in l-arginine group, in the overall cohort; values at baseline (T0—solid pattern) and after 90 days (T1—checkered pattern). Values expressed as median with interquartile range (25°–75° centiles) and min to max. **b** Changes in IIEF-6 score and PSV value over time, from T0 to T1 in l-arginine (continuous green line) compared to placebo (dashed blue line) group, in the overall cohort. Values expressed as median with interquartile range (25°–75° centiles). ***p < 0.0001

**Fig. 3:**
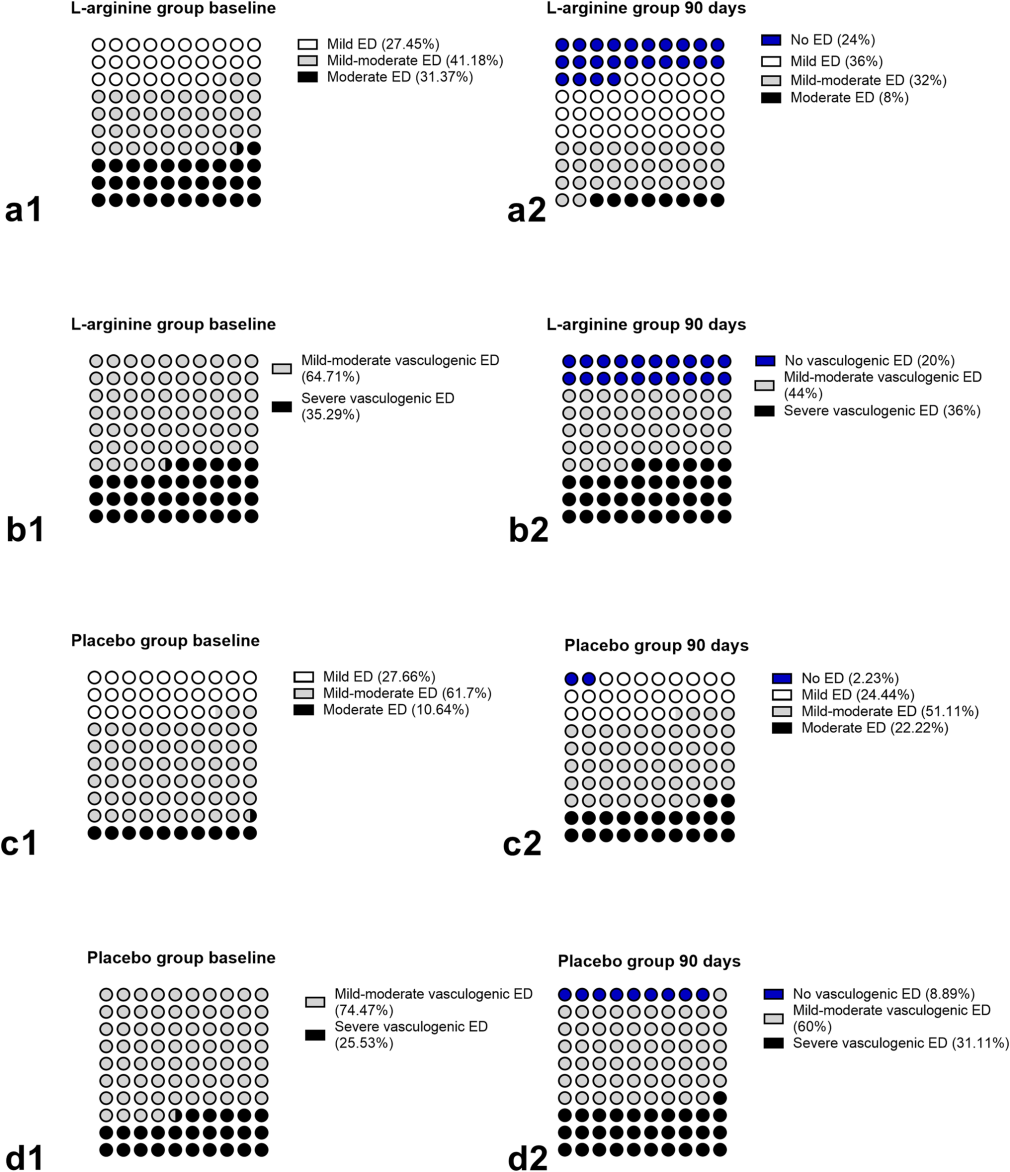
**a** Prevalence of patients without ED (blue dots), and of mild (white dots), mild–moderate (gray dots) and moderate (black dots) ED assessed by IIEF-6 questionnaire in l-arginine group at baseline (a1) vs 90 days (a2). **b** Prevalence of patients without vasculogenic ED (blue dots), and of mild–moderate (gray dots) and severe (black dots) vasculogenic ED assessed at dynamic PDU in l-arginine group at baseline (b1) vs 90 days (b2). **c** Prevalence of patients without ED (blue dots), and of mild (white dots), mild–moderate (gray dots) and moderate (black dots) ED assessed by IIEF-6 questionnaire in placebo group at baseline (c1) vs 90 days (c2). **d** Prevalence of patients without vasculogenic ED (blue dots), and of mild–moderate (gray dots) and severe (black dots) vasculogenic ED assessed at dynamic PDU in placebo group at baseline (d1) vs 90 days (d2)

In l-ARG group, at T1, IIEF-6 score was significantly increased compared to T0 [24 (19–25.3) vs 20 (16–22); *p* < 0.0001] (Fig. 2). Consistently, the prevalence of moderate ED was significantly decreased [4/50 (8%) vs 16/51 (31.37%); *p* = 0.005], with an increase of the prevalence of patients without ED [12/50 (24%) vs 0/51 (0%); *p* = 0.0001], whereas no significant difference was found in the prevalence of mild–moderate and mild ED (Fig. 3). Overall, 37 (74%) patients improved ED degree category, with 12 (24%) patients, mainly belonging to the baseline category of mild ED, reaching IIEF-6 scores compatible with absence of ED; in particular, 8 (50%) and 6 (37.5%) of the 16 patients with moderate ED improved to mild–moderate and mild ED, respectively, whereas 11 (52.38%) and 2 (9.52%) of the 21 patients with mild–moderate ED improved to mild ED or absent ED, respectively, and 10 of the 14 (71.43%) patients with mild ED improved to absent ED. Moreover, 2 (12.5%) patients with moderate ED, 6 (28.57%) patients with mild–moderate ED and 1 (7.14%) patient with mild ED did not change ED degree category. Lastly, 2 (9.52%) patients with mild–moderate ED worsened to moderate ED and 2 (14.29%) patients with mild ED worsened to mild–moderate ED; 1 (7.14%) patient with mild ED dropped out at T1.

**Fig. 4:**
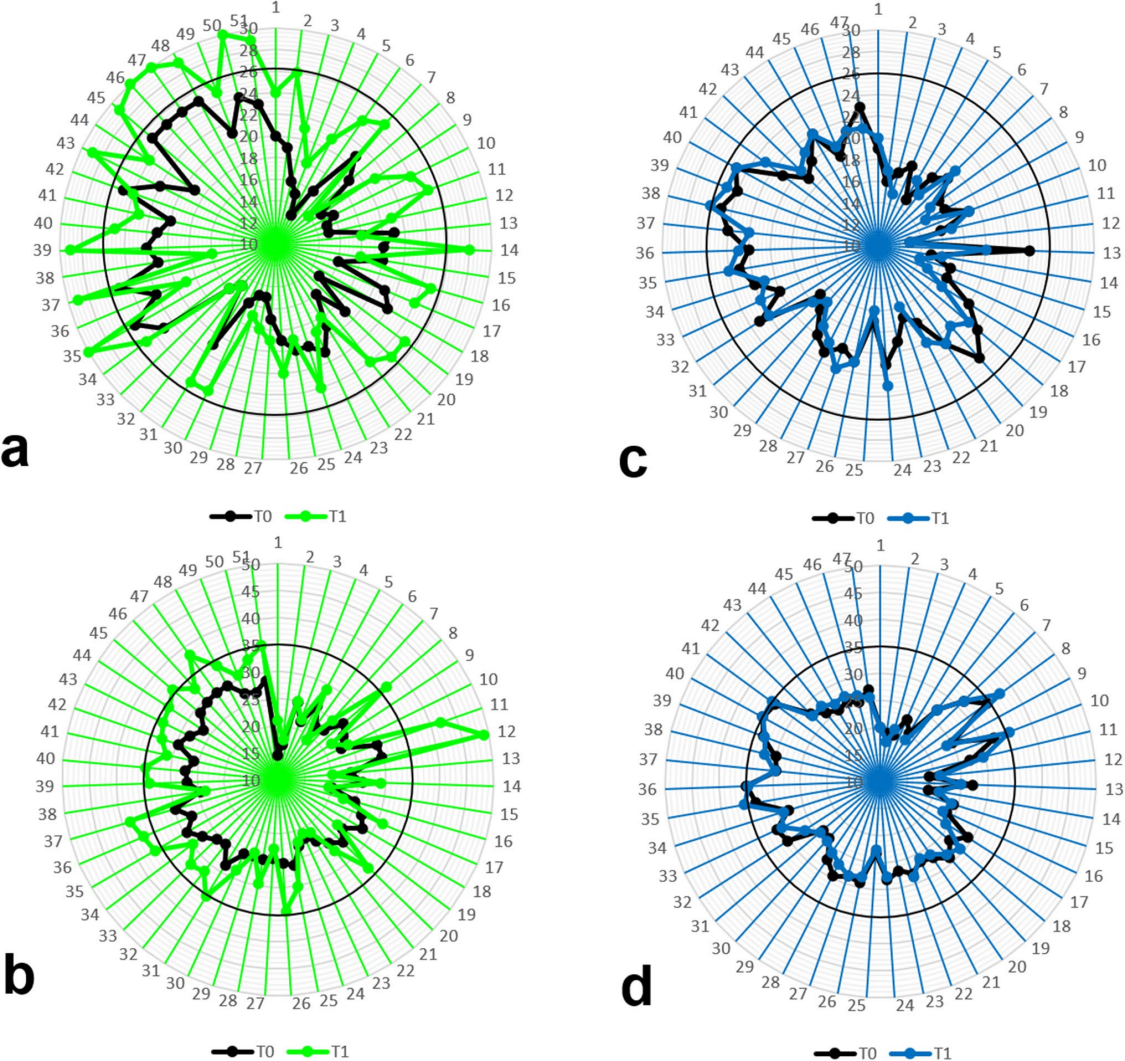
Changes in the ED degree category (**a**) and vasculogenic ED degree category (**b**), from baseline (T0—black series) to 90 days after l-arginine supplementation (T1—green series), for each patient in l-arginine group, and changes in the ED degree category (**c**) and vasculogenic ED degree category (**d**), from baseline (T0—black series) to 90 days (T1—blue series), for each patient in placebo group; the area outside the black circle marks the no ED (IIEF-6 score within the range 26–30 points) category (**a**, **c**) and the no vasculogenic ED (PSV > 35 cm/s) category (**b**, **d**), comprising 12 and 10 patients in l-arginine group, respectively, and 1 and 4 patients in placebo group, respectively, at study completion

In placebo group, at T1, IIEF-6 score was unchanged compared to T0 (Fig. 2). Consistently, no difference was detected in the prevalence of ED degree category (Fig. 3). Overall, 8 (17.78%) patients improved ED degree category; in particular, 3 of the 5 (60%) patients with moderate ED improved to mild–moderate ED, 4 of the 29 (13.79%) patients with mild–moderate ED improved to mild ED, and 1 of the 13 (7.69%) patients with mild ED improved to absent ED. Moreover, 2 (40%) patients with moderate ED, 15 (51.72%) patients with mild–moderate ED and 7 (53.85%) patients with mild ED did not change ED degree category. Lastly, 8 (27.59%) patients with mild–moderate ED worsened to moderate ED and 5 (38.46%) patients with mild ED worsened to mild–moderate ED; 2 (6.9%) patients with mild–moderate ED dropped out at T1. The changes in the ED degree category during the study, for each patient in l-ARG and placebo groups, are shown in Fig. 4.

#### Dynamic PDU

In l-ARG group, at T1, PSV was significantly increased compared to T0 [30.5 (23.4–34.3) cm/s vs 25.9 (24.6–28) cm/s; *p* < 0.0001] (Fig. 2). Consistently, the prevalence of mild–moderate vasculogenic ED was significantly decreased [22/50 (44%) vs 33/51 (64.71%); *p* = 0.047], with an increase of the prevalence of patients without vasculogenic ED [10/50 (20%) vs 0/51 (0%); *p* < 0.001], whereas no significant difference was found in the prevalence of severe [18/50 (36%) vs 18/51 (35.29%); *p* > 0.1] vasculogenic ED (Fig. 3).

Overall, 10 (20%) patients improved vasculogenic ED degree category; in particular, 10 of the 33 (30.3%) patients with mild–moderate vasculogenic ED reached normal PSV values. Conversely, 18 (100%) patients with severe and 22 (66.67%) patients with mild–moderate vasculogenic ED did not change vasculogenic ED degree category; 1 (3.03%) patient with mild–moderate vasculogenic ED dropped out at T1.

In placebo group, at T1, PSV was unchanged compared to T0 (Fig. 2). Consistently, no difference was detected in the prevalence of vasculogenic ED degree category (Fig. 3). Overall, 4 (8.89%) patients improved vasculogenic ED degree category; in particular, 4 of the 35 (11.43%) patients with mild–moderate vasculogenic ED reached normal PSV values. Moreover, 27 (77.14%) patients with mild–moderate vasculogenic ED and 11 of the 12 (91.67%) patients with severe vasculogenic ED did not change vasculogenic ED degree category. Lastly, 3 (8.57%) patients with mild–moderate vasculogenic ED worsened to severe vasculogenic ED; 1 (8.33%) patient with severe vasculogenic ED and 1 (2.86%) patient with mild–moderate vasculogenic ED dropped out at T1. The changes in the vasculogenic ED degree category during the study, for each patient in l-ARG and placebo groups, are shown in Fig. 4.

The relative changes (Δ%) from T0 to T1 in IIEF-6 score (*p* < 0.0001) and PSV values (*p* < 0.0001) were significantly higher in l-ARG compared to placebo groups. The relative changes in the penile erectile function between l-ARG and placebo groups are shown in Table 4.

**Table 4:** Relative changes (Δ%) in penile erectile function from baseline (T0) to 90 days (T1) in l-arginine and placebo groups in the overall cohort, and in the subgroups of patients with baseline mild–moderate or severe vasculogenic ED, assessed at dynamic PDU

	Δ% (T1–T0/T0) l-Arginine	Δ%(T1–T0/T0) Placebo	*p *value
Overall cohort IIEF-6 score	20 (11 to 33.1)	0 (− 5.9 to 5.3)	< 0.0001
Overall cohort PSV (cm/s)	13.8 (− 0.2 to 25.4)	0 (− 2.8 to 5.1)	< 0.0001
Mild–moderate vasculogenic ED subgroup IIEF-6 score	19.5 (14.5 to 31.8)	0 (− 4.9 to 5.3)	< 0.0001
Mild–moderate vasculogenic ED subgroup PSV (cm/s)	23.4 (13.7 to 28.5)	0 (− 2.9 to 5)	< 0.0001
Severe vasculogenic ED subgroup IIEF-6 score	20 (− 6.3 to 42.5)	0 (− 11.8 to 6.3)	0.013
Severe vasculogenic ED subgroup PSV (cm/s)	− 4 (− 8 to 3.1)	0 (− 2.6 to 5.3)	NS

	Δ%(T1–T0/T0) l-Arginine Mild–moderate vasculogenic ED subgroup	Δ%(T1–T0/T0) l-Arginine Severe vasculogenic ED subgroup	*p *value
IIEF-6 score	19.5 (14.5–31.8)	20 (− 6.3 to 42.5)	NS
PSV (cm/s)	23.4 (13.7–28.5)	− 4 (− 8 to 3.1)	< 0.0001

Values expressed as median with interquartile range (25°–75° centiles)

*PSV* cavernous arteries peak systolic flow velocity

### Effect of l-ARG supplementation on penile erectile function according to baseline vasculogenic ED degree

**Fig. 5:**
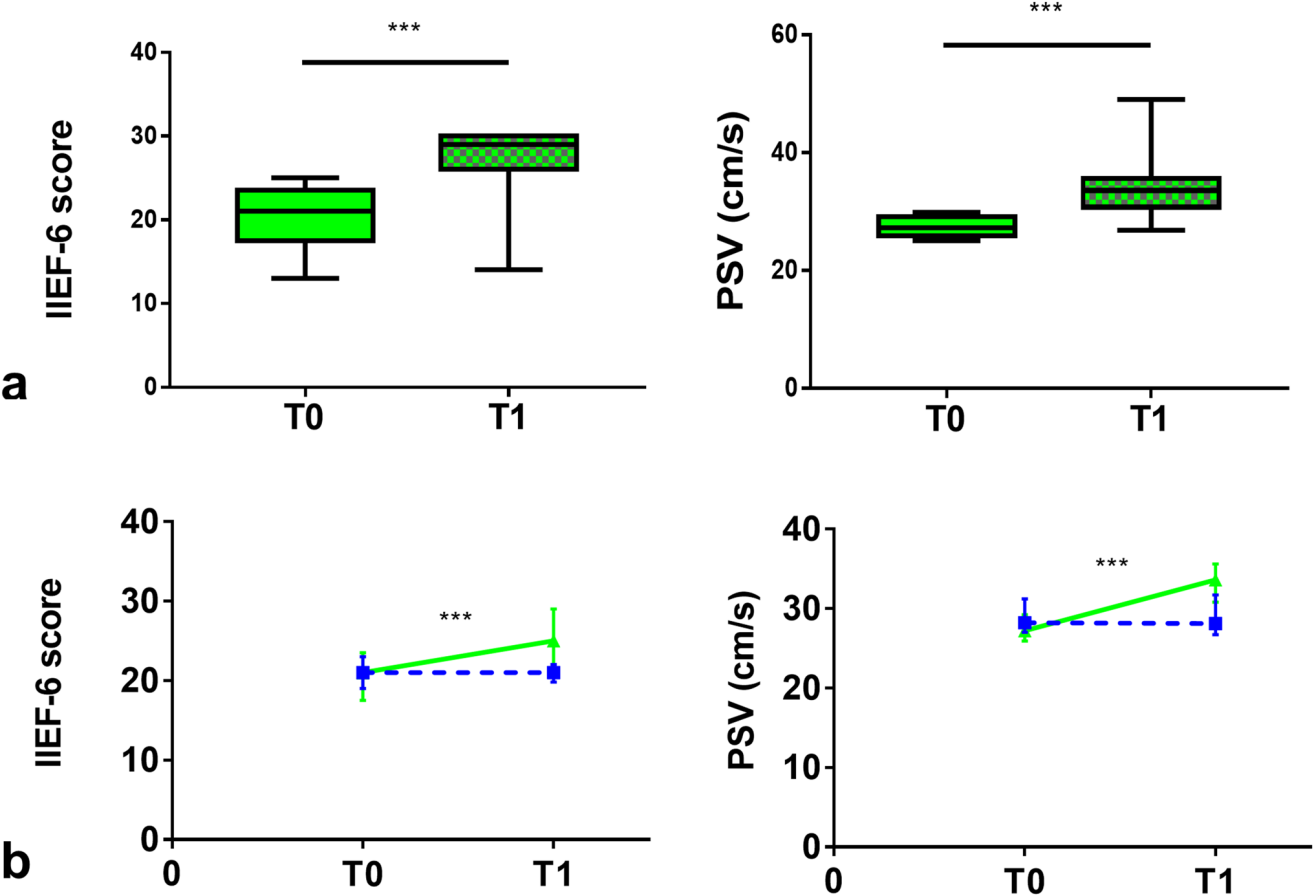
**a** IIEF-6 score and cavernous arteries peak systolic flow velocity (PSV), in l-arginine subgroup of patients with baseline mild–moderate vasculogenic ED assessed at dynamic PDU; values at baseline (T0—solid pattern) and after 90 days (T1—checkered pattern). Values expressed as median with interquartile range (25°–75° centiles) and min to max. **b** Changes in IIEF-6 score and PSV value over time, from T0 to T1 in l-arginine (continuous green line) compared to placebo (dashed blue line) subgroup. Values expressed as median with interquartile range (25°–75° centiles). ***p < 0.0001

In l-ARG subgroup of patients with mild–moderate vasculogenic ED, at T1, both IIEF-6 score [25 (21.3–29) vs 21 (17.5–23.5); *p* < 0.0001] and PSV [33.6 (30.8–35.6) cm/s vs 27.2 (25.9–29.2) cm/s; *p* < 0.0001] were significantly increased compared to T0 (Fig. 5). Conversely, in placebo subgroup of patients with mild–moderate vasculogenic ED, no significant changes were detected in any of the assessed outcome measures. The changes in the penile erectile function during the study in the subgroups of patients with mild–moderate vasculogenic ED are shown in Table 3. The relative changes (Δ%) from T0 to T1 in IIEF-6 score (*p* < 0.0001) and PSV values (*p* < 0.0001) were significantly higher in l-ARG compared to placebo subgroups. The relative changes in the penile erectile function between l-ARG and placebo subgroups of patients with mild–moderate vasculogenic ED are shown in Table 4.

**Fig. 6:**
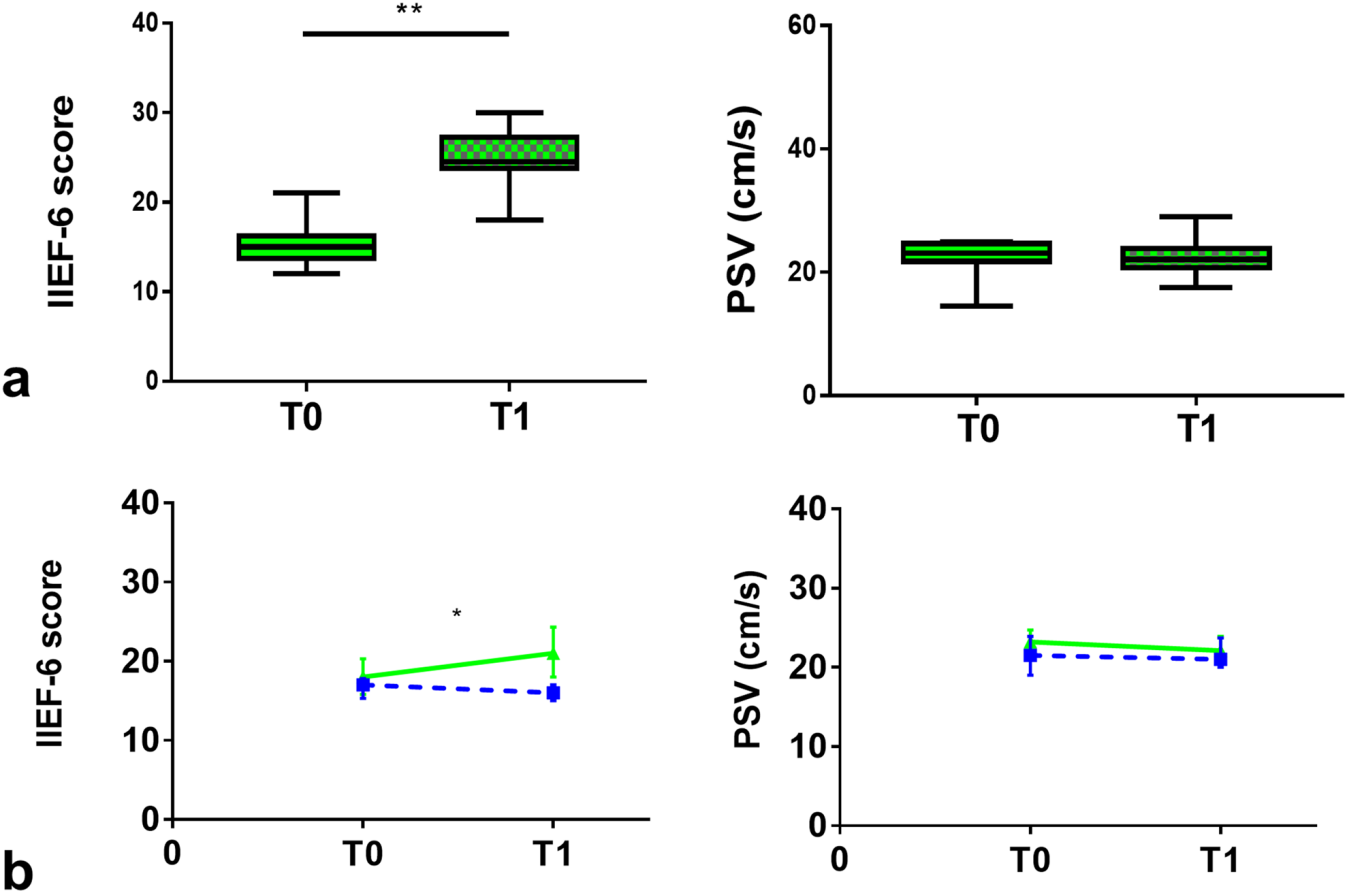
**a** IIEF-6 score and cavernous arteries peak systolic flow velocity (PSV), in l-arginine subgroup of patients with baseline severe vasculogenic ED assessed at dynamic PDU; values at baseline (T0—solid pattern) and after 90 days (T1—checkered pattern). Values expressed as median with interquartile range (25°–75° centiles) and min to max. **b** Changes in IIEF-6 score and PSV value over time, from T0 to T1 in l-arginine (continuous green line) compared to placebo (dashed blue line) subgroup. Values expressed as median with interquartile range (25°–75° centiles). **p = 0.007; *p = 0.013

In l-ARG subgroup of patients with severe vasculogenic ED, IIEF-6 score [21 (18–24.3) vs 18 (15.8–20.3); *p* = 0.007], but not PSV, was significantly increased compared to T0 (Fig. 6). Conversely, in placebo subgroup of patients with severe vasculogenic ED, no significant changes were detected in any of the assessed outcome measures. The changes in the penile erectile function during the study in subgroups of patients with severe vasculogenic ED are shown in Table 3. The relative change (Δ%) from T0 to T1 in IIEF-6 score (*p* = 0.013), but not PSV, was significantly higher in l-ARG compared to placebo subgroups. The relative changes in the penile erectile function between l-ARG and placebo subgroups of patients with severe vasculogenic ED are shown in Table 4.

In l-ARG subgroup of patients with mild–moderate vasculogenic ED, at T1, IIEF-6 score was increased by 19.05% and PSV was increased by 23.53%, compared to T0; in l-ARG subgroup of patients with severe vasculogenic ED, at T1, IIEF-6 score was increased by 16.67% and PSV was decreased by 4.74%, compared to T0. The relative changes (Δ%) from T0 to T1 in IIEF-6 score between mild–moderate and severe vasculogenic ED l-ARG subgroups were not significantly different; conversely, the relative changes (Δ%) from T0 to T1 in PSV values were significantly higher in mild–moderate compared to severe vasculogenic ED subgroups (*p* < 0.0001). The relative changes in the penile erectile function between l-ARG subgroups of patients with mild–moderate and severe vasculogenic ED are shown in Table 4.

### Safety

In l-ARG and placebo groups, no significant changes were detected from T0 to T1, regarding clinical and biochemical parameters, except for HDL cholesterol levels, which were significantly increased in l-ARG group (*p* = 0.001) and decreased in placebo group (*p* = 0.0001). The changes in clinical, biochemical and hormonal characteristics, and in the prevalence of concomitant diseases of patients’ population during the study are shown in Table 5. In l-ARG group, 5.88% of patients experienced adverse events, including gastric pyrosis, urticarial reaction and scrotal itching, none of which was manifested as clinically relevant. In placebo group, 4.26% of patients experienced adverse events, including pyrosis, intense thirst and cholestasis, none of which was manifested as clinically relevant.

**Table 5:** Clinical and biochemical characteristics at baseline (T0) and after 90 days (T1) in l-arginine and placebo groups, in the overall cohort

	l-arginine T0 (*N* = 51)	l-arginine T1 (*N* = 50)	*p* value	Placebo T0 (*N* = 47)	Placebo T1 (*N* = 45)	*p* value	Δ% *p* value^§^
Heart rate (bpm)	73 ± 9	75 ± 9	NS	73 ± 8	73 ± 8	NS	NS
Systolic blood pressure (mmHg)	136 ± 17	133 ± 12	NS	134 ± 12	134 ± 11	NS	NS
Diastolic blood pressure (mmHg)	80 ± 7	81 ± 4	NS	78 ± 9	79 ± 9	NS	NS
Fasting glucose (mg/dl)	102 ± 0.2	100 ± 0.2	NS	106 ± 18	102 ± 14	NS	NS
Triglycerides (mg/dl)	161 ± 97	153 ± 51	NS	159 ± 62	165 ± 58	NS	NS
Total cholesterol (mg/dl)	196 ± 37	192 ± 28	NS	213 ± 37	209 ± 33	NS	NS
HDL cholesterol (mg/dl)	57.9 ± 18.8	60.8 ± 15.6	0.001	58.6 ± 15.4	55.5 ± 13.5	0.0001	< 0.0001
Urea (mg/dl)	36 ± 9	35 ± 8	NS	39 ± 11	39 ± 8	NS	NS
Creatinine (mg/dl)	0.9 ± 0.2	0.9 ± 0.1	NS	0.9 ± 0.2	0.9 ± 0.1	NS	NS
AST (U/l)	25 ± 9	25 ± 7	NS	25 ± 9	26 ± 7	NS	NS
ALT (U/l)	29 ± 12	29 ± 10	NS	30 ± 15	28 ± 10	NS	NS

Values expressed as mean ± SD

^§^Relative changes (Δ%) from baseline (T0) to 90 days (T1) in l-arginine compared to placebo group

## Discussion

Historically, consistent effort was spent in the search of pharmacological and/or more physiological alternatives in the treatment of ED. In this context, growing interest and several research studies have been dedicated to determining the potential role of nutraceuticals in the management of ED, by reporting a variable degree of therapeutic success. Among the nutraceuticals most investigated as single agents, yohimbine has been used for over several decades as a therapeutic option for ED [21], being particularly successful in patients with non-organic forms [22], whereas Korean red ginseng has been suggested to improve ED, although with less firm evidence [23]. The combination of various nutraceuticals and administration of nutraceuticals along with conventional drugs also provided encouraging results, which seem to be superior compared to single agents; the combination of PLC, niacin and l-ARG was demonstrated to significantly improve total and single items of IIEF-6 [15], whereas combined PLC and acetyl-L-carnitine (ALC) was demonstrated to significantly improve penile erectile function together with mood, being more effective than testosterone, in particular, in improving IIEF-6 score and nocturnal penile tumescence in aging men with clinical symptoms of androgen decline [24]. Interestingly, the combination of nutraceuticals with conventional medications currently used for the treatment of ED has been found to potentiate the effect of the medications used as single agents; indeed, the combination of PLC and the PDE5i sildenafil was found to be more effective than sildenafil as single agent in improving penile erectile function in diabetic patients [25], and the combination of PLC, ALC and sildenafil was more effective than sildenafil as single agent in improving IIEF-15 score and the clinical response to PGE1 injection in patients subjected to radical prostatectomy [26].

The results of the current randomized, double-blind, placebo-controlled clinical trial demonstrated that oral administration of l-ARG at the dose of 6 g/day for 3 months significantly improved penile erectile function assessed by IIEF-6 score and PSV evaluated at dynamic PDU in 50 male patients with vasculogenic ED, compared to 45 placebo-treated patients. At study completion, the changes in IIEF-6 score resulted in an overall improvement in ED degree category in 74% of patients, although only 24% patients, mainly belonging to the baseline category of mild ED, reached IIEF-6 scores compatible with absence of ED. Conversely, a minority of patients did not change or even worsened ED degree category. In addition, at study completion, the changes in PSV resulted in an overall improvement in vasculogenic ED degree category in 20% of patients, exclusively belonging to the baseline category of mild–moderate vasculogenic ED, who reached PSV values compatible with absence of vasculogenic ED. Conversely, the totality of patients with severe and the great majority of patients with mild–moderate vasculogenic ED did not change vasculogenic ED degree category. Noteworthy, analyzing results according to baseline vasculogenic ED degree category, at study completion, IIEF-6 score was significantly increased in both subgroups of patients with mild–moderate and severe vasculogenic ED, whereas PSV was significantly increased only in the subgroup of patients with mild–moderate vasculogenic ED, despite a significantly lower baseline PSV in l-ARG compared to placebo group.

The results of the current study are consistent with different reports with partially dissimilar study cohorts and methodologies, demonstrating that daily oral administration of l-ARG improved penile erectile function in men with ED [27, 28]. In particular, one randomized, double-blind, placebo-controlled clinical trial demonstrated that daily oral administration of equal-lower doses (5 g/day) of l-ARG for a shorter period (1 month) compared to the current study significantly increased IIEF-6 score in 34 diabetic middle-aged men affected by mild–moderate ED of unknown etiology [27]. A different randomized, double-blind, placebo-controlled clinical trial demonstrated that daily oral administration of equal-lower doses (5 g/day) of l-ARG for a shorter period (6 weeks) compared to the current study induced a significant subjective improvement of penile erectile function, as assessed through standardized O’Leary questionnaire for ED, although without a significant improvement of penile vascular parameters assessed by basal PDU, in 29 middle-aged/elderly men affected by organic ED of mixed etiology and unknown severity, therefore reporting, in line with the results of the current study, a discrepancy between subjective and objective improvement in erectile function [28]. On the other hand, a study with a different design, namely a randomized, double-blind, placebo-controlled crossover comparison clinical trial, and partially different cohort and methodologies demonstrated that daily oral administration of very low doses (1.5 g/day) of l-ARG for a very short time (17 days) did not improve patients’ satisfaction with their penile erectile function and their sexual life, analyzed by the standardized KEED questionnaire for ED, in 30 middle-aged men affected by ED of mixed etiology and severity [29], suggesting that higher doses and longer-term supplementation might be required, or that l-ARG might be of particular benefit in subtypes of ED, such as those of vasculogenic etiology.

The discrepancies between the results of the various studies might be related not only to the dose of l-ARG or the period of l-ARG supplementation, as well as the population cohort and the study design, but also to the methodology used for the assessment of ED, which was relevantly different among the studies. The assessment of ED in the clinical practice and in clinical trials is based on the administration of different questionnaires specifically validated for the evaluation and/or grading of ED; the aforementioned trials, designed for evaluating the effect of l-ARG supplementation, used standardized O’Leary and KEED questionnaires, addressing the overall sexual function by including erectile and ejaculatory function evaluated during both sexual activity with the partner and masturbatory activity. Nevertheless, the most commonly used questionnaire for investigating sexual function and especially penile erectile function is the IIEF, a validated questionnaire currently used in the majority of clinical trials, with the peculiarity, respect to different questionnaires, not only of distinguishing between presence or absence of ED, but also of grading the severity of ED, in clinical setting [30]. However, IIEF is currently available in 3 different versions: the full version composed of 15 questions (IIEF-15), addressing penile erectile function together with sexual desire, orgasmic function, intercourse satisfaction and overall sexual satisfaction; the 5 questions version (IIEF-5) addressing a partial series of aspects related to penile erectile function together with intercourse satisfaction; and the 6 questions version (IIEF-6) addressing exclusively penile erectile function, but considering the whole spectrum of erection related aspects, including erection frequency, erection firmness, penetration ability, erection maintenance frequency, erection maintenance ability, and erection confidence [30, 31]. The current study used the IIEF-6 questionnaire, favoring its peculiarity of focusing exclusively on penile erectile function, which is the main outcome of the study, despite the limitation of missing additional information on different aspects of sexual function. The choice of IIEF-6 and the preference over the classical IIEF-15 questionnaire was based on the ease and shortness of the test, features potentially increasing patients’ compliance in participating to the assessment of penile erectile function by questionnaire.

The current study also addressed ED by PDU, a second-level diagnostic tool with respect to ED diagnosis, by providing further objective information concerning the penile erectile function, and particularly the vascular status, compared to clinical information deriving from first-level diagnostic tools, such as anamnesis and standardized questionnaires [32–34]. Moreover, in the current study, dynamic PDU was preferred over basal PDU for several orders of reasons: dynamic PDU is more currently used, compared to basal PDU, both in the clinical practice and in clinical trials, for the completion of ED diagnosis and the assessment of erectile response to pharmacological treatments [35]; dynamic PDU is considered by dedicated guidelines as the gold standard for the diagnosis of ED of vasculogenic etiology, by allowing to objectively quantify penile vascular status and identify potential vascular abnormalities, before and during an erection, therefore representing the best candidate tool for the purposes of the current study, focused on vasculogenic ED [35, 36]. Furthermore, dynamic PDU has a crucial role in the differential diagnosis between psychogenic and the most common form of organic ED, namely vasculogenic ED, in patients of different ages [35]; in particular, dynamic PDU has been reported to have a more specific diagnostic value for recognizing or excluding vasculogenic ED in the setting of constitutive severe anxiety-affected young patients, which despite a pathological PSV in flaccid state due to an exaggerated sympathetic tone, might reach a normal PSV value after pharmacologically induced erection, therefore overcoming a potential bias of basal PDU procedure [35–37]. Moreover, in patients with ED, particularly in middle-aged and elderly patients, dynamic PDU may also provide a predictive response for the potential effectiveness of the vasodilative drugs used for ED, as demonstrated by the negative correlation between the severity of penile vascular damage and the clinical response to treatment with PDE5i [38]. Lastly, it is important to remark that ED could represent an early predictor of CV events and the diagnosis of vasculogenic ED at PDU, and particularly at dynamic PDU as the gold standard diagnostic tool, both in patients with or without additional CV risk factors and/or comorbidities, can help to recognize eventual arterial diseases in biggest distal vessels [35, 39–41]. Nevertheless, dynamic PDU is a minimally invasive diagnostic test and might be associated to adverse effects such as mild penile pain and, rarely, in about 5% of cases, priapism, which is mostly spontaneously reversible, and very rarely reversible after prompt therapeutic intervention. Noteworthy, a limitation of the use of dynamic PDU is the contraindication of PGE1 administration in patients with recent CV or cerebrovascular events and with unstable hemodynamic conditions, and its careful administration in patients with risk of arterial hypotension, such as those in treatment with vasodilatory medications, particularly medications increasing NO production [42]; these patients with absolute or partial contraindication to dynamic PDU were indeed initially excluded from the current study.

The current study demonstrated a surprisingly relevant rate of improvement (74%) in ED degree category and a modest rate of improvement (20%) in vasculogenic ED degree category, throughout the entire l-ARG cohort. The great improvement detected in ED degree category should be regarded with caution considering that, in the l-ARG cohort, only 24% of patients, mainly belonging to the baseline category of mild ED, reached IIEF-6 scores compatible with absence of ED, whereas the majority of patients improved the category by remaining within the range of ED. In line with these findings, the modest proportion of patients which improved vasculogenic ED degree category included only those patients belonging to the baseline category of mild–moderate vasculogenic ED, which reached PSV values compatible with absence of ED.

The apparent discrepancy between the proportion of patients improving ED degree category measured by IIEF-6 questionnaire and vasculogenic ED degree category measured at dynamic PDU, might be probably justified by the different characteristics of the two methodologies. Indeed, although IIEF-6 is a validated questionnaire distinguishing between presence or absence of ED by considering the whole spectrum of aspects related to penile erectile function, and also grading the severity of ED in clinical setting, it still remains a subjective tool, prone to the risk of recall bias, patients emotional drawbacks or patients conditioning due to uneasiness or misinterpretation of questions; conversely, dynamic PDU provides a more in-depth appraisal of the underlying penile vascular status and erectile function and an objective measurement of the vasculogenic damage, with the potential limitation represented by inter-operator misalignment, which was overcome in the current study by having all T0-T1 measurements performed by the same clinician, for a given patient.

Moreover, the observed discrepancy in ED degree category improvement according to IIEF-6 or PSV outcomes might be accounted by the absence of patients with baseline severe ED according to IIEF-6 score, as per exclusion criteria, along with the presence of a proportion of patients with baseline severe vasculogenic ED, which were included in the study based on their baseline mild–moderate or moderate ED at IIEF-6 score, as per inclusion criteria; these cohort characteristics might have indeed contributed to widen the amplitude of divergence between the proportion of patients improving ED or vasculogenic ED degree category. In the current study, the differential response to l-ARG supplementation concerning the IIEF-6 score compared to the PSV values is particularly evident in the subgroup of patients with baseline severe vasculogenic ED; the discrepancy between the amelioration in the perceived penile erectile function at IIEF-6 questionnaire and the lack of significant changes in the penile vascular function at dynamic PDU in patients with severe vasculogenic ED might be explained by a potential psychological benefit deriving from treatment, mainly represented by a significant improvement of the subjective perceived quality of penile erection, which was not reflected by an objective improvement of the severe underlying organic disease, a discrepancy occasionally reported in the clinical practice. Moreover, dynamic PDU procedure-related anxiety might occasionally determine an incomplete pharmacologically induced erection, due to the increased sympathetic stimulation and adrenergic tone resulting from situational, non-constitutive, emotional disturbances deriving from the procedure, leading to an incomplete relaxation of cavernous smooth muscle and ensuing decreased blood inflow into the corpora cavernosa of the penis [43]; procedure-related anxiety might be particularly impacting on dynamic PDU outcomes in patients with a severe underlying vascular defect, compared to those with a mild–moderate vascular defect. Therefore, a possible psychological component could not be completely excluded in either direction of effect, namely, a psychological (at IIEF-6 questionnaire) but not objective (at dynamic PDU) improvement, and a negative impact of procedure-related anxiety on dynamic PDU outcomes. Nevertheless, considering the double blinded design of the study, a psychological impact should be symmetrically expected in both l-ARG and placebo groups for both the directions of effect; therefore, it is more plausible that an effective vascular improvement is reached in mild–moderate vasculogenic ED patients, specifically mediated by l-ARG supplementation, as demonstrated by the evidence of PSV values compatible with absence of ED in a subset of mild–moderate vasculogenic ED patients at study completion. This hypothesis might be supported by the assumption that, contrary to severe vasculogenic ED, ED of mild–moderate degree might reflect a less extensive and/or more recently occurred vascular damage, which might be partially reversible and, therefore, more likely to respond to l-ARG supplementation, although this speculation needs objective confirmation.

The rationale behind the administration of l-ARG as a suitable treatment for vasculogenic ED relies on the matter of fact that l-ARG is an important donor of NO, which is a prominent molecular mediator of the penile erectile process, for the ability to induce cavernous smooth muscle relaxation, and particularly to contribute to the cavernous arteries performance and cavernous blood flow, crucial for penis erection [4, 7]. Moreover, NO production in the vascular endothelium of the penis has been found to be decreased in vasculogenic ED, such as in the case of ED caused by atherosclerosis and diabetes [16, 17, 19], and a reduction of circulating l-ARG levels has been detected in patents with vasculogenic ED [20], further confirming that l-ARG supplementation might be beneficial in the treatment of vasculogenic ED.

The major potential molecular explanation for the beneficial effects of l-ARG supplementation on cavernous arteries performance, evaluated through the measurement of PSV at dynamic PDU, observed in the current study especially in the mild–moderate subgroup of patients, might rely on an l-ARG-mediated increase in the NO levels available for the cavernous arteries of the penis. Indeed, in one study with daily oral administration of equal-lower doses (5 g/day) of l-ARG for a shorter period (6 weeks) compared to the current study, l-ARG supplementation was found to significantly increase both plasma and urinary NO levels in patients with organic ED of mixed etiology and unknown severity [28], therefore potentially providing additional intracavernous NO supply; the differential response at dynamic PDU in mild–moderate and severe vasculogenic ED highlighted by the current study might rely on an insufficient intracavernous l-ARG delivery, upon oral administration of l-ARG at the tested doses, unable to counteract a severe vascular impairment, therefore suggesting the potential requirement of higher l-ARG supplementation regimens in these patients, although in some cases of severe vasculogenic ED an irreversible vascular damage cannot be ruled out. A second potentially involved mechanism underlying l-ARG effects on PSV at dynamic PDU might concern l-ARG effect on testosterone levels. Indeed, daily oral administration of equal-lower doses (5 g/day) of l-ARG for shorter period (8 weeks) compared to the current study was found to significantly increase testosterone levels in diabetic patients with mild–moderate ED [16], potentially mediated by increased NO synthesis accompanied by vasodilation and increase of blood flow within the testis, resulting in improved testosterone production. An increase in testosterone production might contribute to the improvement of penile vascular performance and erectile function upon l-ARG supplementation; indeed, testosterone is known to positively influence libido and sexual behavior, and to enhance the activity of penile eNOS and nNOS enzymes, therefore stimulating cavernous smooth muscle relaxation and vasodilation of the cavernous arteries [2, 3]. The hypothesis of an effect of testosterone rise in the l-ARG-mediated improvement of penile erectile function, at least in the mild–moderate vasculogenic ED patients enrolled within the current study, might be partially supported by the evidence of a trend towards increased testosterone levels upon l-ARG supplementation in the overall l-ARG cohort, although testosterone levels were not significantly improved and were already within the normal range at baseline, therefore partially smoothening this hypothesis.

Although ED it is not a life-threatening disease, therapeutic management of ED should not be overlooked, since ED may negatively impact on couple relationship and general health, and even moderate improvements of vascular dysfunction might have a significant clinical impact. Indeed, vasculogenic ED and CV disease (CVD) are considered as different manifestations of a common underlying vascular disorder [4]. Independent meta-analyses concluded that ED may be considered as a predictor of future CVD, particularly coronary heart disease and silent cardiac events [44–47]; this association is particularly relevant in younger men with ED and absence of cardio-metabolic comorbidities, by emphasizing the importance of early diagnosis [4]. Consistently, the Princeton III Consensus Recommendations for the management of ED and CVD indicate that incident ED has a similar or even greater predictive value for CVD, when compared to traditional risk factors exerting a direct detrimental impact on the endothelial function [13]. The endothelium has been widely demonstrated to play a key role in CV pathophysiology, particularly by regulating vascular homeostasis through NO production [19, 48–50]. The oxidative stress, typically observed in pathological conditions determining endothelial dysfunction, might induce endothelial NO depletion and reduced endothelial NO availability, mediated by the interaction of reactive oxygen species with NO to generate oxidative agents potentially damaging cell DNA and proteins, namely reactive nitrogen-containing species [51], or by a direct decrease of NO synthesis, therefore suggesting potential benefit from the administration of NOS substrates, such as l-ARG, as a therapeutic strategy to increase NO production and improve CVD-related endothelial dysfunction [19, 52]. Consistently, human studies demonstrated that l-ARG improved arterial hypertension after both acute intravenous infusion [53] and oral supplementation of l-ARG for 3 months in addition to antihypertensive drugs [54]; moreover, l-ARG has been found to play a relevant positive impact on atherosclerosis, as demonstrated by two RCTs showing that administration of l-ARG improved endothelium-dependent vasodilation of brachial [55] and coronary arteries [56] in patients with angiographically reported coronary artery disease. Therefore, l-ARG supplementation might be considered an attractive approach to ED management in specific settings, such as mild–moderate vasculogenic ED, with an additional potential preventive value for future CVD.

PDE5i currently represent the first-line treatment for ED. The four PDE5i currently approved in both Europe and the United States, and used with variable dosages and formulations, display different pharmacokinetic profiles enabling tailored treatment of ED based on patients requirements; nevertheless, despite all PDE5i provided well-established efficacy results in placebo-controlled RCTs, direct comparison studies among the various PDE5i are scarce, and a clear consensus concerning the most efficient PDE5i is lacking, also due to heterogeneity of methods used to address penile erectile function [57]. A meta-analysis of studies, primarily of short-term (< 12 weeks) duration, and including men with a wide spectrum of comorbid conditions, highlighted an overall percentage of successful sexual intercourse attempts addressed by different tools ranging from 50 to 88% for different PDE5i, compared to around 35% for placebo, therefore reporting a substantial improvement of sexual activity upon PDE5i treatment, and an overall superimposable efficacy of different PDE5i [58]. Despite such a great overall efficacy of PDE5i treatment for ED, some limitations exist, mainly attributable to contraindications and to potential adverse effects and related discontinuation of treatment. Indeed, current guidelines do not recommend administration of PDE5i to patients with unstable angina, severe congestive heart failure, or uncontrolled hypertension, patients at high risk for arrhythmias, and patients receiving nitrates [13]. Moreover, a percentage of patients ranging between 7 and 25%, depending on different dosage, experience adverse events upon PDE5i treatment [59, 60], sometimes manifesting as of moderate or severe entity, the most frequent of which comprise flushing, headache, dyspepsia, back pain, myalgia, dizziness, and rhinitis [10], although discontinuation rates owing to adverse events of PDE5i is generally relatively low, and comprised between 3 and 5% of patients [61]. Lastly, despite PDE5i treatment has been proven a cost-effective therapeutic strategy, compared to no treatment [62], the relatively high cost of a chronic treatment with some reference/equivalent pharmaceutical formulations belonging to this category of drugs might still represent a limitation for patients with economic constraints, by contributing to treatment discontinuation. Overall, these limitations might encourage the search of different approaches, which might combine a more favorable compliance and safety, with acceptable efficacy and with more affordable treatment regimens, and might be therefore particularly suited in a minority of patients experiencing one or more hindrances to PDE5i treatment.

The possible role of l-ARG as an effective alternative for vasculogenic ED therefore relies on different points. First, in clinical practice, it is frequently reported that l-ARG might have a more favorable compliance, since nutraceuticals are perceived as a “more physiological” option, compared to medications. Second, l-ARG supplementation is not associated to specific contraindications, and displays a relatively favorable safety profile; indeed, a systematic review and meta-analysis on different supplements containing l-ARG, as single nutraceutical or in combination, reported that only 2% of patients experienced adverse effects when pooling together only supplements containing l-ARG as single nutraceutical. Moreover, the most frequently experienced adverse effects included headache, itching and insomnia, which were never reported as being severe [7]. Consistently, in the current study, only 5.88% of patients in l-ARG group experienced adverse effects (including gastric pyrosis, urticarial reaction and scrotal itching), but none of which was manifested as clinically relevant, and did not induce treatment discontinuation. These data imply a greater safety of l-ARG, compared to PDE5i, which are not infrequently reported to determine adverse effects, often inducing treatment discontinuation. In line with this evidence, the current study demonstrated a discontinuation rate below 2%, which was lower compared to that reported for PDE5i, and drop-out was unrelated to l-ARG safety profile, being merely determined by patient personal poor confidence concerning the potential benefits of supplementation. Third, l-ARG supplementation is generally less costly than some reference/equivalent pharmaceutical formulations or dosages of PDE5i, therefore being more suitable for long daily treatment regimens. Therefore, l-ARG supplementation as single compound might be considered an attractive alternative approach to ED management in reason of greater compliance and safety profile, and generally more affordable costs, compared to PDE5i.

Interestingly, the combination of l-ARG supplementation with different substances, namely PDE5i, has been reported to induce a greater improvement in penile erectile function, compared to single compounds [16, 17]. In particular, an RCT including 108 diabetic patients with mild–moderate ED demonstrated that a daily treatment with 5 g l-ARG plus 10 mg tadalafil, administered for 8 weeks, significantly improved ED assessed by IIEF-5 and testosterone levels, with a more significant improvement in both endpoints compared to patients receiving tadalafil or l-ARG as single compounds, whereas the significant improvements at basal and dynamic PDU were similar between groups [16]. Consistently, a different RCT including 59 patients with mild–moderate ED of mixed etiology demonstrated that a daily treatment with 3 g l-ARG plus 50 mg of the PDE5i sildenafil, administered for 8 weeks, induced a more significant improvement in IIEF-5, compared to sildenafil as single compound [17]. Therefore, l-ARG supplementation in combination with PDE5i treatment might be considered an alternative therapeutic option to ED management in reason of greater efficacy, compared to PDE5i or L-ARG as single compounds.

The limitations of the current study included the assessment of erectile dysfunction by IIEF-6 rather than IIEF-15 questionnaire, with a consequent potential underestimation of additional domains of sexual function which might have been positively influenced by l-ARG supplementation. Moreover, a relatively small sample size of the subgroup of patients with severe vasculogenic ED might have prevented to capture slightly significant improvements in PSV at dynamic PDU upon l-ARG supplementation, particularly in patients with borderline scores indicating severity, therefore making it challenging to provide conclusive information concerning the potential effect of l-ARG supplementation in patients with borderline severe vasculogenic ED. The current study has several strengths; to the best of our knowledge, the current study is the largest randomized, double-blind, placebo-controlled clinical trial evaluating the effects of l-ARG supplementation as single agent in vasculogenic ED, using a longer-term supplementation period with a relative high dose of l-ARG supplementation. Moreover, this is also the largest randomized, double-blind, placebo-controlled clinical trial with l-ARG supplementation evaluating penile erectile function at dynamic PDU, the gold standard for the diagnosis of vasculogenic ED; to the best of our knowledge, no other studies addressed the effects of l-ARG supplementation in patients with severe ED of any etiology, and, particularly, no studies specifically focused on severe ED of vasculogenic etiology, by excluding different forms of disease.

In conclusion, the results of the current study allow to speculate that l-ARG might represent a potential alternative for patients with mild–moderate vasculogenic ED with contraindication to PDE5i or who had experienced adverse effects upon PDE5i treatment and might require a different therapeutic approach, or, in association to lower PDE5i doses, to reduce the occurrence and severity of PDE5i-associated adverse effects meanwhile potentially exerting an additive or synergistic effect. Moreover, l-ARG might be also generally preferred in patients with economic constraints and in clinical settings in which tolerability should be privileged over efficacy, such as in elderly or polypharmacotherapy. Therefore, l-ARG supplementation might be regarded to as a potential alternative approach in a variety of clinical settings, spanning different therapeutic requirements in the management of ED; nevertheless, additional studies would be suited to further corroborate the encouraging results presented by the current study, and to further widen the field of l-ARG supplementation clinical applicability.

## Data Availability

The datasets generated during and/or analysed during the current study are available from the corresponding author on reasonable request.
